# Dropless after cataract surgery (DACS): concepts, evidence and clinical potential

**DOI:** 10.1038/s41433-026-04604-z

**Published:** 2026-06-26

**Authors:** Johannes Birtel, Ariel Yuhan Ong, Karolin Deichl, Guy Mole, Sher A. Aslam, Peter Charbel Issa

**Affiliations:** 1 Department of Ophthalmology, University Medical Center Hamburg-Eppendorf, Hamburg, Germany; 2 Oxford Eye Hospital, Oxford University Hospitals NHS Foundation Trust, Oxford, UK; 3 Institute of Ophthalmology, University College London, London, UK; 4 Department of Ophthalmology, School of Medicine and Health, TUM University Hospital, Technical University Munich, Munich, Germany

**Keywords:** Lens diseases, Health services

## Abstract

Cataract surgery is one of the most commonly performed surgeries worldwide, and is traditionally followed by a postoperative regimen of eyedrops to reduce the risk of infection and inflammation. However, variable adherence to treatment and the burden of administering drops pose significant challenges. “Dropless after cataract surgery” (DACS) has emerged as an approach where intraoperative delivery may eliminate the need for postoperative drops. DACS offers a simplified alternative to eyedrop therapy, with the potential to improve patient-related quality of life, lower healthcare costs, and promote sustainable practices by reducing medical waste. Multiple studies over the past few decades have demonstrated the effectiveness of intraoperative intracameral antibiotics alone in reducing endophthalmitis rates, thus eliminating the need for topical antibiotic drops. Sustained-release steroid formulations have demonstrated reliable control of postoperative inflammation, similar to those achieved with standard topical regimens, but their adoption remains limited. Realising the full benefits of DACS will require systemic barriers to be addressed, and dropless regimens should be considered within a holistic value-based healthcare approach. As the global burden of cataract surgery continues to grow, embracing dropless techniques may represent a forward-looking step in modernising postoperative management to optimise patient-centred care.

## Introduction

Postoperative care after cataract surgery has traditionally involved the administration of antibiotic and anti-inflammatory eyedrops to reduce the risk of postoperative infection and inflammation. However, this approach has multiple disadvantages, including variable patient adherence and administration techniques, financial and time costs, as well as the environmental impact from single-use plastic containers.

Since the landmark European Society of Cataract and Refractive Surgery (ESCRS) study which established the superiority of intracameral cefuroxime over postoperative topical levofloxacin for endophthalmitis prophylaxis in 2007 [1], treatment practice patterns have gradually shifted. An increasing number of surgeons have opted to replace postoperative topical antibiotics with an intracameral antibiotic solution at the end of surgery, with varying uptake across different countries [2–4]. These findings were supported by a recent international systematic review and meta-analysis of over 1.2 million eyes, which found that both approaches resulted in comparable endophthalmitis rates across a range of real-world studies and randomised controlled trials (RCT) [5]. Further evidence has demonstrated that perioperative antisepsis is more effective than preoperative topical antibiotics in reducing the ocular surface bacterial load [6, 7]. Together, this suggests that topical antibiotics may not have a clear role in preventing infection in routine cataract surgery, and may instead confer a risk of antimicrobial resistance; patients may therefore be best managed with perioperative antisepsis and intracameral antibiotics alone, without the need for postoperative topical antibiotics [6, 8].

Topical corticosteroids and/or non-steroidal anti-inflammatory drugs (NSAIDs) have traditionally been used to control postoperative inflammation following cataract surgery. However, increasing evidence supports the use of sustained-release steroid formulations to provide consistent anti-inflammatory control without the need for postoperative drop administration.

In this review, we discuss the rationale for a “dropless after cataract surgery” (DACS) regimen following routine and uncomplicated cataract surgery. We evaluate several intraoperative alternatives to topical steroids and discuss the evidence base supporting these approaches. In addition, we discuss the patient, clinician, economic, and sustainability perspectives relevant to DACS, and identify systemic barriers and future considerations for broader implementation.

## The evidence base for DACS

Various intraoperative approaches with different routes of administration, formulations, and duration of action have been employed. Options include subconjunctival, sub-Tenon’s, intracameral, and transzonular intravitreal steroid injections, insertion of a steroid-coated intraocular lens, and intracanalicular steroid inserts. Steroid-based strategies remain the most extensively studied (with limited exploration of alternative agents such as NSAIDs). We conducted a PubMed search up to 30 August 2025 for relevant studies of these DACS approaches to inform the narrative review. A modular search strategy which combined core concepts using Boolean operators was employed, the full details of which are presented in Supplementary S1. Only primary research studies were included in the review (Table 1). We provide a summary of their safety and effectiveness here, and conclude with an evaluation of the evidence base.

**Table 1:** Summary of DACS studies in the peer-reviewed literature.

Authors	Country	Year	Study type	Dropless therapy group(s)		Comparator group(s)		Primary outcome measure		Secondary outcome measure(s)	IOP (Dropless vs. comparator)
				Therapy details	N [eyes]	Therapy details	N [eyes]	Parameter	Dropless vs. comparator		
**Subconjunctival triamcinolone acetonide (TA)**											
Shorstein et al. [9]	US	2015	Retrospective	2 mg TA (10 mg/ml or 40 mg/ml)	2061	1% PA eyedrops OR 1% PA eyedrops & NSAID (diclofenac- or flurbiprofen or ketorolac) 4x/day for 4–6 weeks	9255 4754	CMO (CRT thickening with BCVA ≤ 20/40) from day 5–120 after surgery)	No difference in eyes without additional risk factors	Rebound iritis: DL: 3.9% vs. comparator: 5.2%; *P* < 0.05.	Prevalence of IOP spikes ≧ 30 mmHg significantly lower at day 1–3 in DL than in the comparator group (DL: 4.4% vs. comparator: 6.1%, *P* < 0.05);. PA + NSAID group (3.5%) lower than comparator group; *P* < 0.05. Not different between day 4–15 and day 16–45.
Rattan et al. [82]	Iraq	2018	Prospective. Each group allocated to 1 surgeon.	4 mg TA. (10 mg/ml)	500	0.1% dexamethasone eyedrops 4x/day for 1 month	500	AC cells* at day 1, 1 week and 1 month.	Significantly fewer AC cells on day 1 in the DL group. No difference in AC cells at 1 week, 1 and 3 months.	Change in CMT not different at 3 months. Rescue treatment (additional anti-inflammatory eye drops at day 4–21 significantly higher in comparator group): DL: 4.0% vs. comparator: 9.6%.	Increase in IOP ≥ 10 mmHg at 1 month significantly higher in comparator versus DL group (DL: 0.8% vs. comparator: 2.4%).
Lindholm et al. [10]	Finland	2020	Prospective. Non-randomized. Open label.	20 mg TA. (40 mg/ml)	51	0.1% dexamethasone eyedrops 3x/day for 3 weeks	50	Change in CRT, AC flare (flare metre). at days 7, 28 and 90.	Change in CRT significantly higher in the comparator group at all 3 time points. AC flare not different at 7 and 28 days, but significantly less in the DL group at 3 months postop.	No difference in post-OP BCVA, ocular irritation, eye redness and patient satisfaction. Incidence of increase in CRT > 20% significantly lower and duration of eye redness lower in DL group.	IOP not different between groups. No safety concerns reported.
Wu et al. [11]	US	2023	Retrospective	12–20 mg TA. (40 mg/ml)	150 of 112 patients	1% prednisolone eyedrops (4-3-2-1 /day, each tapered down after 1 week)	218 of 173 patients	Proportion of eyes developing steroid response at day 3 to 1 month.	Higher rate of steroid response in the DL group which was not statistically significant (DL:10% vs comparator: 5%, *P* = 0.096; if only one eye/patient is included: DL: 9% vs. comparator: 6%, P = 0.49		Mean IOP after surgery significantly higher and more eyes with prolonged steroid response (> 30 days) in DL-group. No difference in starting IOP-lowering medication (DL: 13% vs. comparator: 12%).
Birtel et al. [83]	UK	2024	Retrospective	3 mg TA. (10 mg/ml)	100	---	---	---	---	CMO in 2%, these patients had also developed CMO in the fellow eye after conventional post-operative management with eye drops. No safety concerns reported.	No cases with increased IOP.
Shorstein et al. [12]	US	2024	Retrospective	4 groups: Low dose (1–3 mg TA) as 10 mg/ml or 40 mg/ml; High dose (3.1–5 mg TA) as 10 mg/ml or 40 mg/ml	11893 (5609, 3890, 1717, 677 respectively)	1% PA acetate. ± NSAID (Diclofenac sodium 1% or ketorolac tromethamine 0.5%)	32509. 25430	Diagnosis of CMO 15–120 days postop. Iritis 15–120 days postop. Glaucoma-related events 15 days-1 year.	CMO: Significantly lower odds in 10 mg/ml high dose group compared to PA group (OR 0.65 (0.43–0.97); *P* = 0.033), trend towards lower odds in other TA groups. Iritis: Not difference across groups	Lower odds of CMO in the 10 mg/ml high dose group compared to PA + NSAID in the post hoc multivariate sensitivity analysis (OR 0.65 (0.42–1.03); *P* = 0.070), but similar odds in the main model. Significantly lower odds of glaucoma-related events in 10 mg/ml low dose group compared to PA group (OR 0.69 (0.55–0.86); *P* = 0.001), higher odds in 40 mg/ml high dose group (OR 2.14 (1.36–3.37); *P* = 0.062), no difference in other TA groups.	Increased odds of elevated IOP in 40 mg/ml high dose TA group.
**Subconjunctival methylprednisolone / betamethasone acetate**											
Dieleman et al. [14]	The Netherlands	2011	Prospective. Randomized. Open label.	5.7 mg/ml betamethasone acetate	200	0.1% dexamethasone eyedrops 3x/day for 4 weeks	200	Foveal centrepoint thickness on OCT. Incidence of CMO (on OCT or clinical significant). AC flare (flare metre) increase from baseline. at 4 weeks.	Foveal centrepoint thickness and CMO: Not significantly different. AC flare: Significantly higher in DL group (*p* = 0.003), but in both groups flare values almost back to pre-op levels (likely not clinically relevant).	No significant difference in additional outpatient clinic visits, additional anti-inflammatory treatment, verbal pain rating, and BCVA. No safety concerns reported. No adverse events.	Not different at day 1 and 4 weeks postop
Merkoudis et al. [15]	Sweden	2014	Prospective, Randomized. Open label.	20 mg methylprednisolone acetate . (40 mg/ml)	25	0.1% dexamethasone eyedrops 3x/day for 2 weeks	25	Increase in IOP at 1 week and 1 month	Not different.	AC cells: Higher grade in topical group (*p* = 0.01) at 1 month. AC flare: Not different at 1 month. No additional anti-inflammatory treatment required in either group (cells did not exceed 1 + ). No safety concerns.	Mean IOP not different at 1 week and 1 month postop.
**Sub-Tenon triamcinolone acetate**											
Paganelli et al. [18]	Brazil	2004	Prospective. Randomized. Double-masked.	40 mg TA	50	1% PA eyedrops (4-3-2-1 /day, each tapered down after 1 week)	50	AC cell score, AC flare score at day 1, 3, 7, 14, 28, 56	Not different. Not different.	No adverse events. No additional treatment needed. No safety concerns reported.	IOP significantly higher in the comparator group at day 3, 7, 14, 28, no difference at day 1 and 56.
Negi et al. [17]	UK	2006	Prospective. Randomized. Masked.	20 mg TA (*n* = 10). 30 mg TA (*n* = 17)	27	0.1% betamethasone eyedrops 4x/day for 30 days	27	AC flare at day 1, 8, 30, 90	Not different.	No difference between groups in frequency of CMO, BCVA and requirement of rescue therapy. No difference in requirement of rescue therapy. No safety concerns reported.	Not different.
Paganelli et al. [16]	Brazil	2009	Prospective, Randomized, Double-masked	25 mg TA. (DuoCat System)	68	1% PA eyedrops 4x/day for 3 weeks	67	AC cells and flare at day 1, 3, 7, 14, 28.	Not different up to 4 weeks	No difference in BCVA and additional anti-inflammatory treatment (1 patient in each group).	Mean IOP change not different.
Khan et al. [19]	Pakistan	2016	Prospective, Randomized.	40 mg TA	54	1% PA eyedrops (4-3-2-1 /day, each tapered down after 1 week)	54	AC cells and flare at day 1, 14 and 6 weeks.	Not different up to 6 weeks	No safety concerns reported.	Not different.
Choopong et al. [20]	Thailand	2022	Prospective. Randomized., Masked.	20 mg TA	70	0.1% dexamethasone eyedrops 4x/day for 4 weeks	70	AC cells and flare; Central foveal thickness at day 1, 7, 14, 28 and 90.	Not different	No difference in BCVA, ocular pain, discomfort and conjunctival redness. No safety concerns reported.	No difference in overall numbers and levels of increased IOP.
Wotipka et al. [21]	US	2023	Retrospective	40 mg TA	234	1% PA eyedrops tapered over 4 to 6 weeks	313	Proportion of BCVA better than 20/40 at 1 month; postoperative complications	Not different.	DL group had slightly higher rates of AC cells, IOP elevation, and early PCO, but the differences were judged to be potentially explained by bias and/or not clinically relevant. AC flare and incidence of CMO not different. No safety concerns reported.	Not different at 1 week and 1 month.
**Sub-Tenon dexamethasone phosphate**											
Erichsen et al. [22]	Denmark	2021	Prospective. Randomized. Open label.	2 mg Dexamethasone phosphate	81	PA 1% + ketorolac 0.5% eye drops—1) both initiated 3 days before surgery, 2) ketorolac initiated 3 days before surgery. Ketorolac 0.5% alone: 3) initiated 3 days before surgery, 4) on day of surgery	85/ 92. 83 / 88	Change in CMT at 3 months	Lower change in CMT in DL group at 3 months (3.5, 98.75% CI –1.1 to 10.1, *p* = 0.04).	Significant differences to comparator group (preop PA and ketorolac) included. Higher change in CMT at 3 weeks and 3 months. Insufficiently controlled inflammation in 48.2% in DL group, relative to 2.3% in control group. Cystoid abnormalities on OCT in 13.3%, relative to 2.3% in the control group. No difference in BCVA at any time point.	Significantly lower IOP in DL group at day 3, but significantly higher IOP in DL group at 3 weeks. IOP at 3 months was not different. Additional anti-inflammatory treatment required in 56.6% (relative to 9.1% in the comparator group).
**Intracameral triamcinolone acetate**											
Karalezli et al. [23]	Turkey	2008	Prospective. Randomized. Open label.	1 mg TA. (1 mg/0.1 ml)	30	1% PA eyedrops (6x/day for 1 week, 4x/day for 15 days)	30	AC cells and flare at days 1, 7 and 30.	Not different.	No difference in BCVA and tolerance (burning, stinging, blurred vision). No safety concerns reported.	Not different at days 1, 7, and 30.
Simaroj et al. [24].	Thailand	2011	Prospective. Randomized. Open label.	2 mg TA. (2 mg/0.1 ml)	30	0.1% dexamethasone eyedrops 4x/day until no inflammation	30	AC cells at day 1, 1 week and 1 month.	Not different in first week, but fewer cells in the TA group at 1 month (*P* = 0.006)	No difference in BCVA at baseline or at 1 month. No adverse events. No safety concerns reported.	Not different at days 1, 7, and 30.
Manzoor & Moin [25]	Pakistan	2018	Prospective. Randomized.	1 mg TA. (1 mg/ 0.1 ml)	30	0.1% dexamethasone eyedrops 4x/day for 4 weeks with gradual tapering	30	AC cells and flare at day 1, 7, 28.	Not different.	No difference in BCVA at baseline, 7 days, or 28 days.. DL group had significantly lower rates of conjunctival irritation and dryness at 1 week and 1 month, but not 3 months.	Not different at days 1, 7, and 28.
Shaheen et al. [26]	Pakistan	2020	Prospective. Randomized.	1 mg TA. (concentration not specified)	40	0.1% dexamethasone eyedrops 4x/day for 4 weeks	40	AC cells and flare at day 1, 7, 28.	Not different.	-	Not different at days 1, 7, and 28.
**Intracameral dexamethasone drug delivery system (DEX DDS; Surodex)**											
Chang et al. [27]	USA	1999	Phase 2. Prospective. Randomized. Double masked.	Dex DDS 60 µg. Dex DDS 120 µg. in AC	28 31	No therapy. Placebo DDS	16 14	Proportion of patients receiving anti-inflammatory rescue medication from day 3 up to 2 months.	DL vs. comparator. Day 3*: 3% vs. 47%; *P* < 0.001. Month 1*: 12% vs. 83%; *P* < 0.001. Significantly less frequent and later in the treatment group at any time point	AC cell and flare scores significantly lower in DL group in the first 3 weeks. Quiet AC at 1 week: DL: 21% vs. comparator: 3%. Safety measures with similar (IOP, BCVA) or better outcomes (less corneal oedema) in intervention group. No safety concerns reported.	Not different.
Tan et al. [28]	Singapore	1999	Prospective. Randomized. Double masked.	Dex DDS 60 µg. in AC	32	0.1% dexamethasone eyedrops 4x/day for 4 weeks	28	AC flare at day 4, 8, 15, 30, 90	Significantly less flare in the DL group in the first month after surgery. No difference after 3 months.	Slit-lamp assessment of AC inflammation: no difference between groups.. Rescue treatment (additional anti-inflammatory eye drops) more frequently required in comparator (*n* = 5) than in DL (*n* = 1) group. No safety concerns reported.	Not statistically compared; no differences described.
Tan et al. [29]	Singapore	2001	Prospective. Randomized. Double masked.	2 Dex DDS 60 µg in AC. 2 Dex DDS 60 µg in PC (ciliary sulcus)	35 36	0.1% dexamethasone eyedrops 4x/day for 4 weeks	33	AC flare (flare metre) at day 4, 8, 15, 30, 90	Significantly less flare in the DL group in the first 2 weeks. No difference after 1 month.	Slit-lamp assessment of AC inflammation at any timepoint: not different. Endothelial cell loss at day 90 and 1 year: not different. Rescue treatment (additional anti-inflammatory eye drops) less frequently required in DL groups (DL: 6% vs. comparator: 27%). Significantly less ocular discomfort, photophobia and lacrimation DL groups. Migration of DDS from ciliary sulcus into AC in 58%.. No safety concerns reported.	Not statistically compared; no differences described.
Wadood et al. [30]	UK	2004	Single-masked parallel group study	Dex DDS 60 µg in AC (saline eye drops postoperative for masking)	11	0.1% dexamethasone eyedrops 4x/day for 4 weeks	8	AC cells and flare at day 1, 3, 7, 14, 21, 28, 60	Not different.	No difference in subjective AC inflammation assessment at any timepoint and in endothelial cell count and BCVA at day 60. Surodex remnants persisted up to a mean of 22 months ± 2.5 (SD) postoperatively in 6 eyes (54%). No severe adverse events. No safety concerns reported.	Not different.
**Intracameral dexamethasone suspension (Dexycu; Eye-Point Pharmaceuticals)**											
Donnenfeld & Holland. [31]	US	2018	Phase 3	342 μg in 5μl 517 μg in 5μl	158. 156	Placebo	80	No AC cells at day 8	High dose (HD): 66.0%. Low dose (LD): 63.1%. Placebo: 25.0%. *P* < 0.001	No AC flare at day 8 (HD / LD / Placebo): 89.1% / 92.4% / 63.8%; *P* < 0.001. No AC flare and cells at day 8 (HD / LD / Placebo): 67.3% / 63.1% / 33.8%; *P* < 0.001. Rescue medications: 7.5%/ 17.5%/ 16.3% in placebo group at day 1, 3, 8; 0%/ 0%/ 1.9% in LD; 0%/ 2.3%/ 1.9% in HD group. Similar rate of adverse events. No serious ocular adverse events.	IOP increase ≥ 10 mmHg (HD / LD / Placebo): 21% / 29% / 13%
Donnenfeld et al. [32]	US	2018	Phase 3	517 μg in 5 μl	126	1% PA eyedrops (4x/day for 3 weeks)	55	Safety (incidence and severity of adverse events) followed up to 90 days	Comparable safety profile between groups, however more adverse events in DL group (DL: 42.1% vs. comparator: 23.6%). Severe adverse event unrelated to study drug. Higher proportion of AC inflammation (DL: 9.5% vs. comparator: 0%) and iritis (DL: 6.3% vs. comparator: 0%) in DL group.	Similar rate of AC cells, AC flare and AC cell clearing up to 90 days (apart from higher rate of AC cell clearing in DL group at day 1). No difference in change of endothelial cell count at day 90. Higher proportion of DL group required rescue medication within first 30 days postop (DL:10.3% vs.comparator: 0%). DL group: 68.7% felt DL surgery was convenient, 67.8% would prefer this in future.	Higher rate of IOP increase in DL group (DL: 11.1% vs. comparator: 3.6%), but only 1 patient required anti-hypertensive therapy.
**Transzonular intravitreal triamcinolone acetate**											
Fisher & Potvin [33]	US	2016	Prospective. Randomized. Subject-masked. Contralateral eye study.	3 mg TA. (Tri-Moxi-Vanc intraocular solution containing 15 mg/ml TA)	25	1% PA + 0.5% Ketorolac eyedrops (3x/day for 1 week, 2x/day for 3 weeks)	25	Change in IOP. At 1 day, 1 week and 1 month.	Not different.	No difference in central corneal thickness (apart from day 1), CMT, BCVA, AC cells and flare, ocular pain and adverse events. Safety: Incidence of rebound inflammation not different between groups (3 in DL and 2 in topical group, p = 0.68). No severe adverse events reported. 92% of patients preferred DL regimen, evaluating the overall surgical experience.	Not different.
Tyson et al. [34]	US	2017	Retrospective	3 mg TA. (Tri-Moxi-Vanc intraocular solution containing 15 mg/ml TA)	1541 (922 patients)	---	---	---	Breakthrough inflammation in 9.2% at days 14-21. Visual significant CMO in 2% at days 14-21 and 90.	Significantly higher rate of breakthrough inflammation at days 14–21 among surgeries performed during the first 7 months of the analysis period compared with those performed in the second 7 months (11.2% vs. 7.8%). Significantly more cases of breakthrough inflammation in patients diagnosed with glaucoma or post-op CMO.. Intraoperative iris prolapse (unknown no) and ciliary body haemorhage. [4]. No case of endophthalmitis, TASS, or HORV.	IOP increase ≥10 mmHg in 0.9% at any timepoint up 90 days.
Singhal et al. [35]	US	2019	Retrospective	3 mg TA. (Tri-Moxi-Vanc intraocular solution containing 15 mg/ml TA)	99 (73 patients)	1% PA eyedrops (4-3-2-1 /day, each tapered down after 1 week)	99 (82 patients)	Breakthrough inflammation requiring anti-inflammatory eyedrops) Between day 1 and 90	DL vs. comparator: 11% vs. 3%; *P* = 0.0488	1/3 of breakthrough inflammation in comparator group due to reported non-compliance. No difference in CMO. No safety concerns reported.	Not different.
Bardoloi et al. [36]	India	2020	Prospective. Non‑randomized.	4 mg TA . (4 mg/0.1 ml Tricort)	196	—	---	Visual acuity at 3 months	BCVA better than 6/9 in all but 1 patient (ERM).	No patients required breakthrough topical steroids. Floaters: 20 patients complained of floaters at day 1, 15 on day 7, 13 on day 30, none on day 60. Learning curve associated with technique—successful injection at first attempt in 70% of cases (defined as seeing a plume of visible suspension within the vitreous)	Normal mean IOP in all cases at all visits.
**Transzonular intravitreal dexamethasone**											
Gounder et al. [38]	India	2024	Prospective. Non‑randomized.	0.4 mg dexamethasone p/f. (4 mg/ml)	50	0.1% dexamethasone 4x/day + 0.1% nepafenac 0.1% 2x/day, all for 4 weeks	50	AC cells and flare*, IOP, BCVA at 1, 3, 7 and 28 days	Significantly lower grades of AC inflammation in DL group from day 1 to day 7 (*p* < 0.001) and no inflammation at day 28 in either group.	Significantly better BCVA in DL group at day 28, but 99% of patients in both groups attained logMAR 0.2 or better. Breakthrough AC inflammation: 3/50 in DL group at day 7, started on topical steroids; none in topical group, but not statistically significant (*P* = 0.24). No safety concerns reported.	No significant difference in IOP throughout the follow-up period.
**Intravitreal triamcinolone acetate via pars plana**											
Haq et al. [84].	US	2020	Retrospective	2.25 mg TA . (Tri-Moxi-Vanc intraocular solution containing 15 mg/ml TA)	571	1% PA 4x/day tapered over 4 weeks + ketorolac 3x/day for 4 weeks	487	Persistent AC inflammation, Rebound AC inflammation, CMO incidence followed up for 6 month (3 and 6 month if available)	DL vs. comparator: 2.6% vs. 8.0%; *P* < 0.001. 2.6% vs. 6.4%; *P* = 0.003. 4.7% vs. 3.9%; *P* = 0.51	DL conferred an increased risk of CMO (OR 3.21, 95% CI 1.42-7.23) but no significant effect on persistent or rebound AC inflammation. Corneal oedema at day 1 or persistent at 1 week (DL: 17.2% vs. comparator: 6%). Ocular discomfort up to 1 month (DL: 2.5% vs. comparator: 0%). Higher incidence of subjective floaters at day 1 in DL (DL: 46% vs. comparator: 10.5%; *P* < 0.001). Higher proportion of DL group had BCVA 20/25 or better (DL: 87.4% vs comparator: 78.8%, *P* = 0.001). Additional anti-inflammatory treatment overall significantly higher in comparator group (DL: 17% vs. comparator: 31.8%).	No difference in overall incidence of IOP increase ≥10 mmHg not different: DL: 1.9% vs. comparator: 0.4%, *p* = 0.027 (reported as not significant). No significant difference in incidence of IOP spike at any other postoperative visit.
**Intracanalicular dexamethasone insert**											
Walters et al. [85]	US	2015	Prospective. Randomized. Double masked.	Dextenza (D) insert 0.4 mg	30	No therapy - Placebo intracanalicular insert	30	Proportion with no ocular pain at day 8. No AC cells at day 8.	DL vs. Placebo. 79.3% vs. 0%; *P* < 0.0001. 20.7% vs. 10.0%; *P* = 0.1495	Absence of AC cells day 30: DL: 62.1% vs. Placebo: 13.8%; *P* = 0.0002. Absence of AC flare day 30: DL: 79.3% vs. Placebo: 27.6%; *P* = 0.025. Rescue therapy day 8 DL: 17.2% vs. Placebo: 55.2%. Adverse events: DL: 13.8% vs. Placebo: 43.3%.	IOP increase ≥10 mmHg in the study eye of 1 patient in each group. Classified as adverse event in each group. IOP increase ≥10 mmHg in the fellow eye of 1 patient in the Dextenza group.
Walters et al. [42]	US	2016	Phase 3	Dextenza (D) insert 0.4 mg	164	No therapy - Placebo intracanalicular insert	83	Proportion with no ocular pain at day 8. No AC cells at day 14.	DL vs. Placebo. 80.4% vs. 43.4%; *P* < 0.0001. 33.1% vs. 14.5%; *P* = 0.0018	Significantly higher proportion without AC flare, and with lower AC cells at days 8 and 14 in Dextenza group. Rescue therapy was required more often in controls than in the DL group at day 8 (DL: 7.4% vs. Placebo: 28.9%) and 14 (DL: 14.7% vs. Placebo: 46.3%). Ease of device insertion: Difficult DL: 7.4% vs. Placebo: 0%. Ocular adverse events occurred more often in PIacebo group (DL: 29.6% vs. PIacebo: 40.5%). No treatment-related serious adverse events in either group.	No significant difference in IOP increase ≥10 mmHg in each group (DL: 6.8% vs Placebo: 3.6%).
Walters et al. [42]	US	2016	Phase 3	Dextenza (D) insert 0.4 mg	161	No therapy - Placebo intracanalicular insert	80	Proportion with no ocular pain at day 8. No AC cells at day 14.	DL vs. Placebo. 77.5% vs. 58.8%; *P* = 0.0025. 39.4% vs. 31.3%; *P* = 0.22	Significantly higher proportion without AC flare, and with lower AC cells at days 8 and 14 in Dextenza group. Rescue therapy was required more often in controls than in the DL group at day 8 (DL: 6.3% vs. PIacebo: 18.8%) and 14 (DL: 17.5% vs. PIacebo: 37.5%). Ease of device insertion: Difficult in DL: 3.1% vs. Placebo: 0%. Ocular adverse events occurred more often in PIacebo group (DL: 28.8% vs. PIacebo: 38.8%). No treatment-related serious adverse events in either group.	No significant difference in IOP increase ≥10 mmHg in each group (DL: 4.4% vs. Placebo: 5%).
Tyson et al. [43]	US	2019	Phase 3	Dextenza (D) insert 0.4 mg	214	No therapy - Placebo insert	221	Proportion with no ocular pain at day 8. No AC cells at day 14.	DL vs. Placebo. 79.6% vs. 61.3%; *P* < 0.0001. 52.3% vs. 31.1%; *P* < 0.0001	Pain, mean AC cells and flare significantly less in the treatment group during the first post-OP month, but not different at day 45. Rescue therapy was required more often in Placebo group at day 14 (DL: 5.6% vs. Placebo 10.9%). Ease of device insertion: Difficult in DL: 2.3% vs. Placebo: 0%. No treatment-related serious adverse events in either group.	IOP increase ≥10 mmHg in 7.4% of DL group (all but 2 at day 2) and 2.7% of PIacebo group; none were judged to be related to treatment and were instead attributed to the cataract surgery.
Donnenfeld et al. [49]	US	2023	Prospective (compared against contralateral eye)	Dextenza (D) insert 0.4 mg & Omidria during surgery (0.3% ketorolac + 1% phenylephrine)	41	1% PA eyedrops (4x/day, for 2 weeks, then 2x/day, for 2 weeks) + ketorolac 0.5% 4x/day, 1 day pre-OP through 4 weeks post-OP	41 (fellow eye)	Proportion with no ocular pain at day 1, 7, 14, 28 and 3 months.	Not different at all time points.	No difference in summed ocular inflammation score (mean of AC cells and flare score) and BCVA. Comparable mean CRT post-op. 94.7% of patients preferred DL management. No adverse events. No difference in side effects/ symptoms.	IOP significantly lower at 1 week in the DL group, but not different at any other time point.

*AC* anterior chamber, *BCVA* best corrected visual acuity, *CMO* cystoid macular oedema, *CRT* central retinal thickness, *DDS* drug delivery system, *DL* dropless, *DuoCat System* triamcinolone combined with Ciprofloxacin in a Controlled-Release System, *IOP* intraocular pressure, *NSAID* non-steroid anti-inflammatory drug, *PA* prednisolone acetate, *TA* triamcinolone.

### Subconjunctival

Subconjunctival triamcinolone acetonide has the largest evidence base of the dropless steroid regimens currently available. A large retrospective cohort study (16,070 eyes) found comparable rates of postoperative cystoid macular oedema (CMO), raised intraocular pressure (IOP), and rebound iritis for patients treated with subconjunctival triamcinolone (2 mg) compared to standard topical treatments (1% prednisolone with or without adjunctive NSAID drops) in eyes without additional risk factors for developing these complications [9]. A small study comparing patients treated with a higher dose of subconjunctival triamcinolone (20 mg) against topical steroids reported comparable IOP in both groups [10]. However, another study involving similarly high doses (12–20 mg) suggested that factors such as glaucoma may confer an increased risk of raised IOP with subconjunctival triamcinolone [11]. More recently, a large retrospective study (57,939 eyes) compared different lower doses (1–3 mg or 3.1–5 mg) and concentrations (10 mg/ml or 40 mg/ml) of subconjunctival triamcinolone to standard topical treatments (1% prednisolone with or without adjunctive NSAID drops) [12]. The authors reported comparable odds of postoperative inflammation across all groups, with significantly lower odds of postoperative CMO in the 10 mg/ml high dose group. Although the authors did not specifically examine IOP risk in patients with glaucoma, they evaluated glaucoma-related events (a composite outcome including IOP spike, IOP elevation, and a new diagnosis or worsening of glaucoma or ocular hypertension), and found significantly lower odds of developing such events in the 10 mg/ml low dose group, with higher odds in the 40 mg/ml high dose group.

The optimally safe and effective dose of subconjunctival triamcinolone would ideally be investigated in a high-quality RCT. The ESCRS-funded EPICAT study is a European multicentre RCT which evaluates subconjunctival triamcinolone (10 mg) as a dropless treatment strategy. Preliminary results suggest that it is safe and effective. Rates of postoperative CMO and IOP spikes were comparable to the control group (topical steroids and NSAIDs), and were significantly better than other treatment arms such as intracameral ketorolac alone [13]. The final results may help to shape guidelines and support broader adoption across ESCRS member countries.

Other subconjunctivally administered agents have also been evaluated. Two RCTs compared subconjunctival betamethasone acetate (5.7 mg) [14] and subconjunctival methylprednisolone (20 mg) [15] with topical steroid regimens. Neither reported clinically significant differences in anterior chamber (AC) inflammation and IOP between the intervention and control groups at four weeks postoperatively, suggesting that both treatment regimens were safe and effective.

### Sub-Tenon

In terms of sub-Tenon steroid delivery, several small prospective RCTs (54-140 eyes) have compared sub-Tenon triamcinolone acetate (20–40 mg) to topical steroids and found no difference between the two groups, albeit with AC flare rather than clinically relevant measures as the primary outcome [16–20]. A larger retrospective study (547 eyes) showed higher rates of AC inflammation and early posterior capsular opacification in the sub-Tenon triamcinolone group, which the authors judged to be related to bias from the study design influencing differential surgical complexity across both groups. Importantly, the differences in AC inflammation were not clinically significant and did not affect visual acuity [21].

Another RCT compared sub-Tenon dexamethasone phosphate 2 mg with topical steroids and/or NSAIDs with or without preoperative treatment initiation [22]. Despite comparable visual acuity outcomes, close to half of the sub-Tenon group had insufficiently controlled inflammation requiring additional topical treatment and postoperative visits, suggesting that sub-Tenon’s dexamethasone 2 mg was inadequate for these purposes. Whether a higher treatment dose will be effective remains unclear, but IOP control appeared adequate up to 3 months postoperatively at this dose.

### Intracameral steroid delivery

Three steroid formulations (triamcinolone acetate, dexamethasone drug delivery system, and dexamethasone suspension) have been tested for intracameral steroid delivery.

Intracameral injection of triamcinolone acetate (1 mg or 2 mg) has been assessed in small RCTs of 30-40 patients each [23–26]. Intracameral triamcinolone was as effective in controlling postoperative inflammation as topical steroids, and no significant differences were reported in terms of safety, IOP, or visual outcome.

The efficacy of an intracameral dexamethasone drug delivery system (DEX DDS), which is similar to Ozurdex® but smaller in size and targeting the anterior segment, has also been assessed in 3 small double-masked RCTs [27–29] and 1 single-masked parallel group study [30] ranging from 19 to 89 patients in total. The drug delivery system is a biodegradable device that allows sustained drug release after anterior chamber insertion, and contains 60 μg of microdispersed dexamethasone (equivalent to one drop of dexamethasone 0.1% ophthalmic suspension). DEX DDS was safe and effective in reducing intraocular inflammation after cataract surgery, and the intervention group required rescue treatment less frequently than the control arm [28, 29]. No inter-group difference in IOP or endothelial cell loss was detected. Administering a second DEX DDS did not reduce aqueous flare, and the insertion site (AC or ciliary sulcus) did not influence therapeutic efficacy [29]. Despite the promising performance, it was never introduced onto the market for anterior segment delivery.

Intracameral dexamethasone suspension (342 and 517 µg) is a slow-release formulation that was evaluated in two multicentre double-masked RCTs, one of which compared this against a standard topical steroid regimen, with the other using placebo as a comparator [31, 32]. AC cells, flare, and cells plus flare clearance was similar to a control group receiving topical steroids, and lower than in the placebo control group. In both studies, the treatment group featured a higher proportion of patients with increased IOP, although the authors reported comparable safety across groups.

### Transzonular injection

Triamcinolone acetate may also be administered as a transzonular injection into the vitreous cavity, where it is given in combination with moxifloxacin with or without vancomycin (as ‘Tri-Moxi-Vanc’ or ‘Tri-Moxi’). Effectiveness and safety (in terms of IOP control) has been assessed in large retrospective studies with or without a control group [33–38]. However, the rates of breakthrough inflammation requiring additional topical treatment appeared to be higher than expected—9.2% in a large uncontrolled study of 1541 patients [34], and 11% in the treatment group compared to 3% in the control group in a retrospective cohort study of 198 eyes [35]. In addition, patients have reported floaters and cloudy vision with Tri-Moxi in the early postoperative period. This relates to the opaque nature of the combination of drugs used (moxifloxacin and triamcinolone acetonide), and can be particularly bothersome to certain patients [39]. This risk – and the transient nature of any vitreous debris—should be discussed during the informed consent process. Concerns have been raised about the potential risks of transzonular injections in eyes with compromised zonular support (e.g. pseudoexfoliation, trauma) or which have been vitrectomised [39], although no specific adverse events related to such patients have been reported thus far.

It is important to note here that intraocular vancomycin has been associated with haemorrhagic occlusive retinal vasculitis, a rare but visually devastating condition. Consequently, its use is no longer recommended in routine cataract surgery by professional organisations such as the American Academy of Ophthalmologists [40, 41], although it remains a valuable treatment option for postoperative endophthalmitis, where the benefits outweigh the risks.

### Intracanalicular depot

Another option is Dextenza®, a sustained-release intracanalicular dexamethasone depot containing 0.4 mg preservative-free dexamethasone which delivers treatment to the ocular surface for up to 30 days. Its safety and efficacy has been evaluated in several multicentre phase 3 clinical trials (range: 60–435 eyes), in which absence of pain at day 8 and absence of AC cells at day 14 were designated as the primary efficacy endpoints [42, 43]. Compared to controls (vehicle only), there was a statistically significant difference favouring the Dextenza® group in terms of absence of ocular pain, AC cells, and the need for breakthrough treatment with topical steroids. The adverse events reported were mild and transient. Only a few surgeons (3.1–7.4%) reported difficulties with insertion [42], which may represent a learning curve that can be remedied with time.

### Other innovations

Researchers are currently exploring drug-eluting intraocular lens implants (IOL) which turn the IOL into a sustained-release therapeutic device by attaching a drug reservoir or coating [44–46]. While a fair amount of effort has been expended in developing these drug delivery systems and optimising the materials and methods for delivery over the last few decades, clinical translation has been slower to follow [44]. Nevertheless, early prototypes involving a polymer ring attached to the IOL haptic have demonstrated promise in delivering NSAID therapy to reduce pain, and prevent inflammation and CMO in a phase 1 study involving five patients [47]. Other work exploring drug-containing microspheres attached to the IOL is ongoing; beyond reducing the risk of postoperative inflammation, the ability to control drug release also has potential applications to other fields such as glaucoma care [48].

Additional steroid-sparing alternatives in the form of intracameral NSAIDs are also being explored. One study evaluated a combination of intracanalicular Dextenza® and intracameral phenylephrine 1%/ketorolac 0.3% compared to topical steroid therapy in a prospective within-subject trial, with comparable safety and efficacy outcomes observed [49]. Further work by the same authors has demonstrated better postoperative ocular pain control and reduced intraocular inflammation for a nepafenac punctal plug delivery system compared to a placebo delivery system [50].

## Considering the evidence base for DACS

Overall, the evidence base shows that DACS is safe and effective in the post-operative management of uncomplicated cataract surgery, relative to traditional treatment with eyedrops. There are several options with different routes of administration and formulations supported by different levels of evidence. For example, phase 3 trials were necessary to support the regulatory approval of novel drug delivery systems and devices (e.g. intracameral dexamethasone drug delivery system, intracameral dexamethasone suspension, intracanalicular dexamethasone insert), and sample sizes may be limited by cost and resource constraints. Conversely, in situations where drugs can be administered without the need of an additional delivery system (e.g. subconjunctival triamcinolone acetonide), large retrospective real-world studies with or without a comparator arm dominated. While RCTs are typically seen as the gold standard for generating evidence in many fields of medicine, there is significant value in real-world evidence, which tends to be more reflective of real-world practice as well as patients’ treatment adherence to eyedrops in the comparator arm.

Although primary outcome measures tended to vary across studies, common measures including safety indicators (e.g. IOP elevation), markers of effectiveness (e.g. AC cells and flare, requirement for rescue topical steroids), and more subjective patient-centred outcomes such as pain were typically preferred depending on the study type. Patient-reported outcome measures were otherwise lacking. Clinically relevant outcomes such as postoperative CMO were not always described. In addition, there was a lack of direct head-to-head comparisons between different formulations and modes of administration, and the variable outcomes, follow-up intervals, and exclusion criteria make direct comparisons difficult. Finally, while most studies compared the DACS intervention against standard care (topical treatment with different combinations of steroid and/or NSAIDs and/or antibiotics), several trials (including many of the intracanalicular dexamethasone insert trials) used placebo treatment as a comparator, which may not enable a fair comparison of safety or effectiveness. Overall, the development and adoption of a core outcome set (COS) for assessing DACS (for example, building on the COS defined for standard cataract surgery [51]) would support more consistent and meaningful evaluation of these interventions.

Considerations for the “optimal” treatment strategy in terms of formulation, drug, dose, and administration route will most likely depend on specific procurement costs, reimbursement strategies, sufficiency and quality of evidence, regulatory approvals, and drug availability in each hospital and healthcare system (Table 2). While several have been approved for commercial use by the US Food and Drug Administration (FDA), none of these have been authorised by regulators in other jurisdictions such as the European Union (EU) or Australia (Table 2). Others such as Tri-Moxi or Tri-Moxi-Vanc require a specialised pharmacy to perform compounding.

**Table 2:** Summary of DACS steroid options.

Route of administration	Drug	Regulatory status*	Comments
Subconjunctival	Triamcinolone acetonide	Off-label use	Strongest evidence base and real-world adoption. Long-term evidence for the treatment of ocular inflammatory disease
	Methylprednisolone	Off-label use	Long-term evidence for the treatment of ocular inflammatory disease
	Betamethasone acetate	Off-label use	Long-term evidence for the treatment of ocular inflammatory disease
Sub-Tenon	Triamcinolone acetonide	Off-label use	Long-term evidence for the treatment of ocular inflammatory disease
	Dexamethasone phosphate	Off-label use	
Intracameral	Triamcinolone acetonide	Off-label use	
	Dexamethasone drug delivery system (DEX DDS, Surodex®)	FDA approved; not MHRA, EMA or TGA approved	Cost. Sustained release
	Dexamethasone suspension (Dexycu®)	FDA approved; not MHRA, EMA or TGA approved	Cost. Slow release suspension
Transzonular intravitreal	Triamcinolone acetonide (given as Tri-Moxi or Tri-Moxi-Vanc)	Off-label use (patented but not yet FDA/ MHRA/ EMA/ TGA approved)	Needs to be compounded; risk of floaters.
	Dexamethasone	Off-label use	
Intracanalicular	Dexamethasone insert (Dextenza®)	FDA approved; not MHRA or EMA approved	Cost. Sustained release

*Regulatory information accurate as of April 2025.

*DACS* dropless after cataract surgery, *FDA* Food and Drug Administration (USA), *MHRA* Medicines & Healthcare Products Regulatory Agency (UK), *EMA* European Medicines Agency (Europe), *TGA* Therapeutic Goods Administration (Australia).

Currently, subconjunctival triamcinolone has the strongest evidence base in terms of large real-world studies that comprehensively assess clinically relevant treatment outcomes. Although used off-label in the context of cataract surgery (as are most DACS alternatives), it has a long history of safe and effective use in managing ocular inflammatory diseases, also on an off-label basis. RCT evidence, including findings from the EPICAT study [13], is forthcoming and will provide further data on its role in postoperative inflammation control.

Finally, careful patient selection is also key, as patients with intraoperative complications or a history of uveitis may require more intensive or adjustable postoperative treatment. Patients at higher risk of postoperative uveitis, prolonged or rebound postoperative inflammation (e.g. intraoperative pupil expansion, ethnic backgrounds tending towards more heavily pigmented irides) [52], or cystoid macular oedema (e.g. those with diabetes or significant epiretinal membranes) may also be less suitable for fixed-dose DACS treatment instead of tailored topical treatment. Similarly, patients with ocular comorbidities such as glaucoma, prior steroid-induced IOP elevation, or other conditions requiring close postoperative monitoring may benefit from the flexibility of treatment modulation offered by topical treatment rather than a fixed-dose injection with a sustained-release steroid. More research is vital to investigate these patient groups in detail. Notably, the risk of IOP elevation may also vary depending on the mode of delivery—intracameral delivery of triamcinolone and dexamethasone has not been linked with IOP elevation, probably as a result of the rapid aqueous humour turnover (~1% of AC volume per minute) [53], albeit with relatively comparable rates of AC inflammation postoperatively. In contrast, cases with elevated IOPs have been reported in some studies of sustained-released methods such as subconjunctival, sub-Tenon, transzonular intravitreal triamcinolone, or the slow-release formulation of intracameral dexamethasone suspension. This means that the choice and implementation of DACS regimens should also account for ocular history, anticipated postoperative inflammatory response, and the need for therapeutic flexibility.

## The patient perspective

DACS may confer multiple advantages for patients and their carers by simplifying postoperative care, removing the need for treatment adherence, reducing the associated caregiver burden, and preserving patient comfort.

Dropless regimens at the point of care remove the risk of postoperative human error, adherence issues, and delays in treatment initiation engendered by logistical issues in obtaining the eyedrops. With traditional topical treatment, treatment success often relies on both adherence and accurate instillation to achieve maximum therapeutic effect. However, adherence to postoperative treatment regimens can be variable – electronic compliance monitoring studies have found a mean dose compliance of 50% and premature treatment discontinuation in patients after cataract or glaucoma surgery [54]. Administration is often complicated by surgeons prescribing one to three eyedrops postoperatively, with variable dosing intervals and sometimes a tapering regimen of topical steroids that further complicate adherence [55]. Extensive data from patients with glaucoma and dry eye disease corroborate these findings, demonstrating that difficulties with administration, inconvenience, and behaviour and lifestyle factors are significant barriers to treatment with eyedrops [56–58]. In addition, many patients who persist with treatment find this challenging–in one study, up to 93% of eyedrop-naive patients demonstrated improper administration techniques, and there was a large discrepancy between patients’ perceptions of their efforts and clinicians’ observations of their techniques [59]. Related problems include bottle contamination due to poor hand hygiene or contact with eyelids/eyelashes with the dropper tip, or trauma to the cornea or conjunctiva (affecting up to 68% of elderly patients) [60]. This may become even more challenging in patients with limited dexterity, tremors, poor vision, cognitive impairment or intellectual disabilities, who may require additional support from caregivers to manage their postoperative regimens.

Topical medications also carry the risk of ocular surface toxicity which can cause irritation and discomfort for the patient, and may necessitate further visits to healthcare providers for treatment.

As such, qualitative evaluation suggests that patients may appreciate the DACS model. Patients participating in a within-subject RCT comparing an intracanalicular dexamethasone insert (Dextenza®) and topical steroids tended to prefer the former (up to 95%) [49]. A smaller real-world cross-sectional survey reported that 93% of patients preferred this to topical steroid drops [61], while another suggested that a high proportion (96%) enjoyed the convenience afforded by the dropless treatment, and that 92% would recommend this experience to friends and family [62].

## The clinician perspective

From the clinician’s perspective, beyond considerations regarding the safety and effectiveness of dropless regimens, other factors such as personal preference may play a role as well. Surgeons may be less comfortable with novel formulations and modes of administration such as intracanalicular inserts or transzonular intravitreal injections, preferring established topical regimens. Other surgeons may prefer the flexibility of adjusting treatment (for example, extending the treatment course or adjusting the frequency) instead of a fixed-dose one-off treatment. Yet others may prefer a regimen combining steroids and NSAIDs, and the latter has not been thoroughly investigated in DACS research to date.

However, DACS may hold significant potential to streamline postoperative cataract care pathways and improve resource efficiency for clinicians. Postoperative eyedrop regimens entail a significant administrative burden—improper instillation techniques may necessitate further prescriptions, queries from patients about difficulties with drops in terms of side effects and need for further prescriptions, and phone calls from pharmacies seeking clarification [61]. By simplifying the postoperative process, clinicians and support staff can spend less time on medication counselling and administrative tasks, allowing valuable resources to be re-allocated to other needs. This has the potential to improve staff satisfaction as well—as a small cross-sectional survey on the real-world experience of DACS regimens attests, 95% of practice staff were satisfied with the marked reduction in time spent educating patients on postoperative treatment and fielding phone calls from pharmacies [61].

## The economic perspective

The economic benefits of DACS can be considered in terms of direct and indirect costs to the patient and the healthcare system. This includes the financial costs of the medication itself, as well as the need for additional support to administer topical treatment, both of which will vary across different healthcare systems.

Dropless regimens can potentially provide indirect economic benefits by reducing the need for carer support in patients who are unable to self-administer topical treatment, which may have indirect downstream effects on economic productivity. For those without carer support, socialised healthcare systems such as the UK’s National Health Service (NHS) may employ district nurses to conduct home visits to perform this task. The following costs are all projected for 2025: the travel and salary costs of a single visit from a district nurse approximate £22.10, which totals £2475 if four visits are required each day for the full 28-day postoperative treatment regimen [63]. In addition, there is limited district nurse capacity which could otherwise be re-allocated for other tasks in an already over-stretched healthcare service.

In terms of direct medication costs, in the UK, a subconjunctival steroid depot of triamcinolone acetonide (Adcortyl®) costs the NHS £3.63, which is comparable to the costs of topical treatment with preserved dexamethasone 0.1% drops (£2.84), but is less costly than dual treatment with both dexamethasone 0.1% and ketorolac 0.5% (£7.98) which is preferred by some surgeons. This is significantly less costly than the preservative-free form of dexamethasone 0.1% (£20.96–£62.88) (Supplementary S2) [64]. Overall, cost savings can potentially still be significant given that half a million cataract surgeries are performed in the UK per year, and savings from DACS may increase once indirect costs are taken into account [65].

For comparison, the cost difference of a single subconjunctival injection (€2.16) versus preserved dexamethasone 0.1% eye drops (€15.58 for two 5 ml bottles) in Germany is €13.42. If prednisolone acetate 1% eye drops are used (€31.96 for two 5 ml bottles), cost savings from DACS increase to €29.80. The discrepancy grows significantly further for preservative-free preparations (Supplementary S3). Extrapolated to roughly 900,000 cataract surgeries performed annually in Germany, these direct savings amount to between €12 million and €75 million per year, depending on the comparator used, corresponding to approximately a 7- to 39-fold difference. Importantly, these calculations consider the least expensive topical option, which is not necessarily the most frequently prescribed option in clinical practice, suggesting that real-world savings could be even greater. Moreover, these figures do not yet account for potential indirect savings, including a reduced need for caregiver support, which can vary considerably across different regions in Germany.

For insurance-based healthcare systems such as the US, DACS has the additional benefit of reducing the financial strain on patients by eliminating out-of-pocket medication costs. A 2016 report found that intraoperative anti-inflammatory and antibiotic administration cost approximately USD$100 compared to USD$350 for a standard postoperative eyedrop regimen, meaning potential savings of USD$250 per eye, or USD$13 billion for the period 2016-2025 if a dropless regimen was adopted [66]. A more recent retrospective cost analysis estimated that a dropless regimen comprising intracameral moxifloxacin and subconjunctival triamcinolone acetonide would result in national healthcare and out-of-pocket cost savings of USD$131.63 per eye when compared to the least expensive topical regimen, potentially saving up to USD$675 million per annum [67].

The overall cost-effectiveness of DACS will vary depending on the structure and funding mechanisms of each healthcare system. Comprehensive economic evaluations across diverse healthcare systems (including cost-effectiveness models and real-world data) are essential to support these theoretical considerations, in addition to taking broader societal and operational perspectives into account.

## The sustainability perspective

DACS may confer additional benefits of contributing to sustainability in healthcare. Surveys involving close to 1500 ophthalmic surgeons across the US and Europe reveal an almost unanimous agreement on the importance of reducing surgical waste [68, 69]. Patients also showed strong awareness of anthropogenic climate change and the need for ophthalmology to become more sustainable [70]. Efforts to improve environmental sustainability in ophthalmology are underway, including multi-dose drop recycling initiatives and the reduction of single-use supplies for high-volume procedures such as cataract surgery or intravitreal injections [71–74]. DACS may contribute to these initiatives by reducing the need for medication bottles and packaging. While the savings for each procedure are modest, the aggregate impact appears substantial – eliminating two or three drop bottles for millions of surgeries worldwide per annum will lessen landfill load and reduce the production of plastic and chemicals for eyedrop bottles, which translates to significant environmental benefit overall. This may also reduce patients’ need for additional trips to pharmacies to refill prescriptions or to clinics for drop-related issues, indirectly cutting down on travel emissions and therefore reducing the carbon footprint associated with each surgery.

Although current considerations point towards a favourable environmental profile for DACS, a comprehensive assessment of its sustainability will benefit from detailed life cycle analyses (LCA) [75].

## Barriers to adoption and considerations for mitigation

While DACS has been routinely performed in a large integrated managed care consortium in the US which has offered subconjunctival triamcinolone in place of topical steroid drops since 2008 [12] and in several other areas (e.g. a few NHS trusts and independent sector providers in the UK), uptake has otherwise been slow. Evidence on adoption across different countries is limited as well.

Unlike the ESCRS study, which demonstrated clear superiority of intracameral cefuroxime over placebo or topical antibiotics [1], most dropless steroid regimens are non-inferior to topical postoperative steroids, which may fail to generate the same impetus for practice change. Institutional inertia may also be fuelled by concerns over the risk of errors from drug compounding or preparation, which are necessary for more cost-effective non-commercial DACS options that do not come pre-prepackaged in a drug delivery system, and which are typically used on an off-label basis.

The lack of a cohesive endorsement from professional organisations exacerbates this uncertainty, leaving undecided clinicians without clear guidance on whether dropless approaches should replace the current standard of postoperative care. Clear evidence-based guidelines or recommendations from professional bodies or societies may therefore be helpful in allaying surgeons’ concerns about potential medicolegal issues arising from switching to DACS regimens. As of early 2026, the UK and Ireland Society of Cataract and Refractive Surgeons (UKISCRS) society has led the way in issuing recommendations supporting DACS in appropriate patient populations, citing its robust evidence base in the management of uncomplicated cataract surgeries [76]. At the same time, robust consenting processes should be considered to allay any residual concerns regarding adoption. This includes documenting the clinical rationale and patient counselling. Under the Montgomery standard, patients must be informed of material risks that a reasonable person in their position would consider significant as well as the available alternatives. This includes the off-label nature of commonly used intracameral or periocular agents, the limited long-term comparative data, the small risk of IOP rise and limited reversibility and titratability once DACS is administered (as opposed to eye drops which can be tailored), and the option of continuing with a traditional topical regimen should they prefer this.

Reimbursement policies may present additional obstacles, particularly in insurance-based healthcare systems such as the US. However, innovations such as intracanalicular dexamethasone inserts have secured distinct current procedure terminology (CPT) codes that allow for reimbursement and potentially easing the path to adoption. In Europe, reimbursement decisions fall to individual national health (technology) assessment bodies (such as NICE, HAS, or G-BA), each with different evidentiary thresholds, creating a fragmented landscape where adoption in one country offers no guarantee of access in another. In addition, in many publicly funded systems, injectable medications are not reimbursed separately when considered an integral part of a ‘dropless’ procedure [77], reducing the financial incentives to adopt dropless approaches. Such financial disincentives are short-sighted and overlook broader systemic benefits such as reduced follow-ups for drop-related issues or complications from non-adherence to treatment. Shifting financial incentives from discrete procedural elements toward total episode-of-care value can better support models like DACS that offer such downstream benefits, which are typically invisible in traditional reimbursement paradigms.

As such, aligning DACS with value-based healthcare (VBHC) initiatives and incorporating this into bundled payment frameworks may therefore be key to accelerating uptake [78, 79]. In addition, performance-based incentives linked to quality metrics such as patient-reported outcome measures, postoperative complication rates, or unplanned healthcare utilisation could also highlight the relative advantages of DACS and reinforce its role within broader VBHC initiatives. Achieving this will require coordinated multi-stakeholder collaboration from clinicians, patients, professional bodies, regulators, and payers to build a consensus framework that captures both clinical and health economic value to support sustainable implementation at scale.

Although a DACS regimen can offer several potential advantages, implementation has to be guided by careful patient selection and an understanding of associated limitations. DACS may be less suitable for patients with known or suspected steroid-induced ocular hypertension or glaucoma with poor IOP control due to the potential need for adjusting anti-inflammatory therapy. Similarly, patients with uveitis or other conditions prone to exaggerated inflammatory response may benefit from adjustable topical regimens rather than fixed-dose implants or injections. Intraoperative complications may also necessitate intensified postoperative monitoring or tailored topical treatment. In addition, sustained-release steroid depots may be unsuitable in patients with significant conjunctival scarring or altered ocular anatomy, where predictable drug delivery is uncertain.

## Conclusion

The postoperative cataract surgery pathway has changed significantly over the past three decades. For instance, day one postoperative reviews were once de rigeur, but are no longer standard practice in countries such as the UK and France, although this practice persists in other countries [80, 81]. Similarly, DACS has the potential to reimagine postoperative care by simplifying the postoperative regimen, with potential benefits for patients, carers, clinicians, healthcare systems, and the environment. A growing evidence base demonstrates the safety and effectiveness of DACS following routine cataract surgery. However, uptake has been modest thus far. To advance adoption, future work should involve development of clear clinical guidelines from professional societies, and implementation will need to be tailored to the structure and priorities of individual healthcare systems, considering factors such as surgical reimbursement models, patient preferences and acceptability, and medication pricing.

Supplemental material is available at Eye’s website

## Supplementary information

Supplement
