# Supplementary material for: Dropless after cataract surgery (DACS): concepts, evidence and clinical potential
Source: Eye (Lond). 2026 Jun 26;40(11):1602–17. doi: 10.1038/s41433-026-04604-z (PMC13415846; doi:10.1038/s41433-026-04604-z)
Supplement: Supplementary file 1 — Supplement [file 41433_2026_4604_MOESM1_ESM.docx]

**Supplementary Materials**

**Supplementary S1: Pubmed search strategy used in this narrative review.**

("Cataract Extraction"[MeSH]  OR "cataract surgery"[tiab] OR "phacoemulsification"[tiab])

AND

 ("dropless"[tiab]

OR

 (("intracameral"[tiab] OR "subconjunctival"[tiab] OR "transzonular"[tiab] OR "sub-Tenon"[tiab] OR "intraocular"[tiab])

AND

("steroid"[tiab] OR "triamcinolone"[tiab] OR "dexamethasone"[tiab] OR "antibiotic"[tiab] OR "anti inflammatory"[tiab])))

**Supplementary S2: Cost comparison of a single subconjunctival injection of triamcinolone acetonide and postoperative drop regimens after cataract surgery using preserved dexamethasone 0.1% eyedrops and preservative free dexamethasone 0.1% eyedrops in the UK. Costs taken from the 2022 NHS Business Services Authority (NHSBSA) tariff book.**

|  | **Cost per unit** | **Total cost for a 4x/day 4-week regimen** |
| --- | --- | --- |
| **Subconjunctival triamcinolone acetonide 10mg/ml** | £3.63 | £3.63 for one-off administration |
| **Topical Dexamethasone 0.1% – preserved** | £1.42/bottle | £2.84 for 2 bottles |
| **Topical Dexamethasone 0.1% – preservative-free** | £10.48/box, each containing 20 single-dose containers | £62.88 for 6 boxes if using a single container for each administration (manufacturer recommendations)  £20.96 for 2 boxes if using a single container for each 24-hour period |
| **Topical Ketorolac 0.5%** | £3.99/bottle | £7.98 for 2 bottles |

​​

**Supplementary S3: Cost comparison of a single subconjunctival injection of triamcinolone acetonide and postoperative drop regimens after cataract surgery using preserved dexamethasone 0.1% eyedrops, preservative free dexamethasone 0.1% eyedrops and preserved prednisolone acetate 1% eyedrops in Germany. Costs taken from the Rote Liste (Arzneimittelverzeichnis), May 2025. SDU, single-dose unit.**

|  | **Costs per unit**  **(range depending on manufacturer)** | **Total cost for a 4x/day 4-week regimen (112 drops total)** |
| --- | --- | --- |
| **Subconjunctival triamcinolone acetonide 10mg/ml** | €2.16 per 1 ml ampoule (based on a 100-unit box) | € 2.16 for one-off administration |
| **Topical Dexamethasone 0.1% - preserved** | € 14.60 - 21.62/bottle (5ml) | € 15.58*- 43.24 for 2 bottles |
| **Topical Dexamethasone 0.1% - preservative-free** | € 16.28 - 23.30 per box (10 SDUs)  € 20.58 - 23.26 per box (20 SDUs)  € 31.61 - 50.78 per box (50 SDUs) | €83.80 - 124.92 for 2 boxes of 50 SDUs and one box of 20 SDUs (total: 120 SDUs), if using a single container for each administration (manufacturer recommendations)  €36.86 - 46.66 for one box of 10 SDUs and one box of 20 SDUs (total: 30 SDUs), if using a single container for each 24-hour |
| **Topical Prednisolone Acetate 1% - preserved** | € 15,98 - 19,97/bottle (5ml) | € 31.96 - 39.94 for 2 bottles |

*Dexafluid 1 mg/ml (MIBE GmbH), discounted when prescribed as a 2x5 ml box.

# 
